# Magnesium Hydride-Mediated Sustainable Hydrogen Supply Prolongs the Vase Life of Cut Carnation Flowers via Hydrogen Sulfide

**DOI:** 10.3389/fpls.2020.595376

**Published:** 2020-12-09

**Authors:** Longna Li, Yuhao Liu, Shu Wang, Jianxin Zou, Wenjiang Ding, Wenbiao Shen

**Affiliations:** ^1^Laboratory Center of Life Sciences, College of Life Sciences, Nanjing Agricultural University, Nanjing, China; ^2^Center of Hydrogen Science, Shanghai Jiao Tong University, Shanghai, China

**Keywords:** magnesium hydride, hydrogen gas, hydrogen sulfide, vase life, cut carnation flowers

## Abstract

Magnesium hydride (MgH_2_) is a promising solid-state hydrogen source with high storage capacity (7.6 wt%). Although it is recently established that MgH_2_ has potential applications in medicine because it sustainably supplies hydrogen gas (H_2_), the biological functions of MgH_2_ in plants have not been observed yet. Also, the slow reaction kinetics restricts its practical applications. In this report, MgH_2_ (98% purity; 0.5–25 μm size) was firstly used as a hydrogen generation source for postharvest preservation of flowers. Compared with the direct hydrolysis of MgH_2_ in water, the efficiency of hydrogen production from MgH_2_ hydrolysis could be greatly improved when the citrate buffer solution is introduced. These results were further confirmed in the flower vase experiment by showing higher efficiency in increasing the production and the residence time of H_2_ in solution, compared with hydrogen-rich water. Mimicking the response of hydrogen-rich water and sodium hydrosulfide (a hydrogen sulfide donor), subsequent experiments discovered that MgH_2_-citrate buffer solution not only stimulated hydrogen sulfide (H_2_S) synthesis but also significantly prolonged the vase life of cut carnation flowers. Meanwhile, redox homeostasis was reestablished, and the increased transcripts of representative senescence-associated genes, including *DcbGal* and *DcGST1*, were partly abolished. By contrast, the discussed responses were obviously blocked by the inhibition of endogenous H_2_S with hypotaurine, an H_2_S scavenger. These results clearly revealed that MgH_2_-supplying H_2_ could prolong the vase life of cut carnation flowers via H_2_S signaling, and our results, therefore, open a new window for the possible application of hydrogen-releasing materials in agriculture.

## Introduction

Hydrogen is an ideal energy carrier that is being increasingly used in both power generation applications and transportation. Besides, hydrogen gas (H_2_) has been documented having a range of biological effects and gradually utilized in medicine and agriculture (Ohsawa et al., 2007; Xie et al., 2012; Zeng et al., 2013; Wu et al., 2019). Clearly, the storage of hydrogen is one of the key challenges in developing a hydrogen economy. The storage methods include pressurized gas, a cryogenic liquid, and solid fuel as chemically or physically combination with materials, such as metal hydrides (Sakintuna et al., 2007). At present, the supplementation of H_2_ for biological research includes a gas cylinder and water electrolysis, and H_2_ is normally dissolved in water and saline (Ohta, 2011; Xie et al., 2014; Li et al., 2018; Su et al., 2018). However, the extensive application of the hydrogen-rich liquid solution is limited due to the low solubility and short residence time of H_2_ in water. Fortunately, the growing development of solid hydrogen-storage materials may provide ways to improve the issues about production and storage of H_2_, considering portable, safety, large hydrogen contents, and sustainable hydrogen supply of solid-state storage (Hirscher et al., 2020).

Magnesium hydride (MgH_2_) stands as a promising hydrogen source because of its high hydrogen-storage capacity (7.6 wt%), abundant resources, and low cost (Grochala and Edwards, 2004). The research on applications of MgH_2_ and its related compounds has focused on thermal storage for solar power stations and hydrogen supply for vehicles (Bogdanović et al., 1995; Schlapbach and Zuttel, 2001; Baricco et al., 2017; Lototskyy et al., 2018; Hirscher et al., 2020). It is well documented that MgH_2_ can produce a desired quantity of H_2_ by the following hydrolysis reaction at room temperature: MgH_2_ + 2H_2_O → Mg(OH)_2_ + 2H_2_, the by-product of which is environmentally friendly. This property of MgH_2_ makes a possible for biological application. Amazingly, Kamimura et al. (2016) discovered that orally given MgH_2_ could increase the content of blood H_2_ and decrease the level of plasma triglyceride in rats, thus extending their average lifespan. These results indicated that MgH_2_ with biosafety might also have potential roles in medical applications.

In fact, there are two disadvantages of MgH_2_ restricting its further practical application: (1) the reaction kinetics of MgH_2_ hydrolysis is extremely slow in pure water; (2) the insoluble layer of magnesium hydroxide [Mg(OH)_2_] rapidly coated on the outer surface of the unreacted MgH_2_ to further hide reaction as the pH increases (Hiraki et al., 2012). Subsequently, some organic acids (including citric acid, ethylenediamine-tetraacetic acid, and tartaric acid) were found as good buffer agents to effectively accelerate the reaction, finally improving H_2_ generation by decreasing the pH and suppressing Mg(OH)_2_ formation (Hiraki et al., 2012; Chao, 2018). On the other hand, it is well-known that organic acid-induced decrease in pH of vase solutions inhibits bacterial growth and increases the water conduction in the xylem of cut flowers, thus prolonging the vase life (van Doorn, 2010).

The postharvest senescence of cut flowers results in significant commercial losses, which is closely associated with a series of signaling molecules, including ethylene (Kumar et al., 2008), reactive oxygen species (ROS; van Doorn and Woltering, 2008), nitric oxide (NO; Naing et al., 2017), and hydrogen sulfide (H_2_S; Zhang et al., 2011). Highly coordinated changes in gene expression are also involved (Shahri and Tahir, 2011). Many senescence-associated genes (*SAGs*) have been cloned from carnation petals, and their expression patterns were examined as well. For example, transcripts of representative genes encoding β-galactosidase (*DcbGal*) and glutathione-*S*-transferase (*DcGST1*), previously described as *SR12* and *SR8*, are increased during flower senescence (Lawton et al., 1989; Meyer et al., 1991).

Recently, the usage of H_2_ in the form of hydrogen-rich water (HRW) was observed to delay postharvest senescence and improve the quality of cut flowers (Ren et al., 2017; Su et al., 2019; Wang et al., 2020). Subsequent biochemical analysis showed that H_2_ prolonged the vase life of cut rose and lily was mediated by maintaining water balance, increasing antioxidant defense, and prolonging cell membranes stability (Ren et al., 2017). Meanwhile, H_2_ can inhibit ethylene synthesis and corresponding signal transduction via regulating the expressions of related genes (such as ethylene synthesis genes *Rh-ACS3* and *Rh-ACO1* and ethylene receptor genes *Rh-ETR1*), thus delaying rose senescence during the vase period (Wang et al., 2020). In addition, H_2_-stimulated NO, another gaseous molecule, can act as a downstream signal molecule involving keeping postharvest freshness in cut lily (Huo et al., 2018). However, the effects of sustained hydrogen supply on prolonging the vase life of cut flowers and related mechanisms are still elusive.

In this study, we firstly aim to find an optimized condition for using MgH_2_ in the flower vase experiment. It was confirmed that the application of citrate buffer solution (CBS) could greatly accelerate the reaction rate of MgH_2_ hydrolysis, confirmed by the rapid and sustainable increased H_2_ generation, thus showing more efficiency in the residence time of H_2_ in solution, compared with HRW. By using pharmacological and molecular approaches, we discovered that the combined treatment of MgH_2_ and CBS could remarkably prolong the vase life of a cut carnation flower, compared with either treatment with MgH_2_ or HRW, or CBS alone. It is a new finding. Further results suggested that the discussed MgH_2_-CBS response is mediated by influencing H_2_S signaling. Together, this work will not only extend the application of MgH_2_ to agricultural practices but also provide a new idea for the development of new plant growth regulators.

## Materials and Methods

### Chemicals

All chemicals used in our experiments were purchased from Sigma-Aldrich (St. Louis, MO, United States) unless stated otherwise. MgH_2_ was obtained from the Center of Hydrogen Science, Shanghai Jiao Tong University (Ma et al., 2019). MgH_2_ was further characterized by using scanning electron microscopy (SU-8010, Hitachi, Tokyo, Japan), X-ray diffraction (D/MAX-Ultima III, Rigaku, Tokyo, Japan) with Cu K radiation source, differential scanning calorimetry (STA449F3, Netzsch, Selb, Germany), and thermogravimetry (TG209F3, Netzsch, Selb, Germany). In addition, sodium hydrosulfide (NaHS) and hypotaurine (HT) were used as an H_2_S releasing compound and a specific H_2_S-scavenger, respectively (Ortega et al., 2008). H_2_S fluorescent probe 3-oxo-3H-spiro[isobenzofuran-1,9’-xanthene]-3’,6’-diyl bis(2-(pyridin-2-yldisulfanyl)benzoate) (WSP-5; MKBio, Shanghai, China) was used to monitored endogenous H_2_S in cut flowers (Peng et al., 2014). The concentrations of these chemicals were selected based on the results of pilot experiments.

### Plant Material and Treatments

Cut carnation “Pink Diamond” flowers at the typical commercial stage (the petals form a right angle with the stem axis) were purchased from a flower market in Nanjing City, Jiangsu Province, China, from July to September of 2019. They were transported within 1 h to the laboratory. Subsequently, the cut flower stems were placed in distilled water and re-cut underwater to a length of 25 cm. The top two leaves were kept as well.

The cut flower stems were incubated in glass bottles with 150-ml distilled water (control) and 0.1-M CBS (pH 3.4) containing 0.01, 0.1, and 1 g L^–1^ MgH_2_. Because the treatment with 0.1-M CBS (pH 3.4) plus 0.1 g L^–1^ MgH_2_ showed the most obvious effects on prolonging the vase life of a cut flower in a pilot experiment (Supplementary Figures 1A–C), this combined treatment was applied subsequently. Meanwhile, 0.1 g L^–1^ MgH_2_, 0.1 M CBS (pH 3.4), or 10% HRW (obtained by water electrolysis) alone was, respectively, regarded as controls, and HRW was prepared according to the previous method (Su et al., 2019).

To confirm the possibility that the effect of MgH_2_ was only due to molecular hydrogen and not associated with magnesium ion, MgH_2_-CBS solution was boiled for three times, 5 min each to remove the generated H_2_, followed by keeping under the normal temperature condition for 1 day until no H_2_ was detected.

Because 600-μM NaHS and 10-mM HT showed the obviously promoting and repressing effects on prolonging the vase life of a cut flower in pilot experiments, respectively (Supplementary Figures 1D,E), these treatments were also chosen. For further tests, the cut flower stems were incubated in treatment solutions (150 ml) containing distilled water (control), 0.1 g L^–1^ MgH_2_-CBS, 600-μM NaHS, or 10-mM HT, alone and in combination. For the entire tests, all stems were continuously kept in the treatment solutions throughout the vase period at 25 ± 2°C, 60–70% relative humidity, and 12 h per day of light (20 μmol m^–2^ s^–1^). All treatment solutions were renewed daily as well.

### Determination of Hydrogen Gas Concentration

The concentration of H_2_ in solutions was measured by a portable dissolved hydrogen meter (ENH-1000, TRUSTLEX, Osaka, Japan) that was calibrated by gas chromatography (Su et al., 2019).

### Vase Life, Relative Fresh Weight, and Flower Diameter

The vase life of each flower was calculated as the number of days from the day that the stems were placed in the vase solutions (recorded as day 0) until the day that 50% of petals had wilted or the stems had bent (bent-neck angle greater than 45°). During the vase period, the fresh weight of each sample was measured daily using an analytical balance. The relative fresh weight (RFW) was calculated as following: RFW% = (FW_t_/Fw_0_) × 100, where W_t_ is the fresh weight of the sample (g) at day t (t = 0, 1, 2, 3, etc.), and W_0_ is the fresh weight of the same sample (g) at day 0. Additionally, flower diameter was defined as the maximum width of each flower and measured daily using a digital caliper. In each experiment, 10 flowers were placed per treatment with three replications, and the means of the vase life, RFW, and flower diameter were determined.

### Measurement of Endogenous Hydrogen Sulfide

With the aid of laser scanning confocal microscopy, H_2_S level *in vivo* was determined as described previously with minor modification (Kou et al., 2018). The petals were incubated with 20-μM WSP5 (an H_2_S fluorescent probe) in 20-mM 4-(2-hydroxyethyl)-1-piperazineethanesulfonic acid–sodium hydroxide buffer (pH 7.5) for 30 min in the dark (25°C). After three washes (10 min per time) with fresh 4-(2-hydroxyethyl)-1-piperazineethanesulfonic acid–sodium hydroxide buffer, the samples were observed using an LSM 710 microscope (Carl Zeiss, Oberkochen, Germany) with excitation at 495 nm and emission at 525 nm. The bright-field images were shown at the lower right corners of their corresponding fluorescent images. The relative fluorescence was presented as relative units of pixel intensities calculated by the ZEN software to the control samples. At least five sections per sample were determined, and three samples in each treatment were used.

### Histochemical Staining and Corresponding Measurement of Hydrogen Peroxide Content

The hydrogen peroxide (H_2_O_2_) in petal was visually detected according to the method of Thordal-Christensen et al. (1997). The petals were stained with 0.1% 3,3-diaminobenzidine for 12 h at room temperature in the dark. Afterward, the petals were detected under a light microscope (Stemi 2000-C; Carl Zeiss, Germany).

The H_2_O_2_ content was measured by the spectrophotography (Mei et al., 2017). The samples were incubated with assay reagent (containing 50-mM H_2_SO_4_, 200-μM xylenol orange, and 200-mM sorbitol) for 45 min in the dark at 25°C. Then, the absorbance values were determined at 560 nm. A standard curve was obtained by adding a variable amount of H_2_O_2_.

### Analysis of Senescence-Associated Genes Transcription

Quantitative real-time RT-PCR (qPCR) was used to analyze the expression of *SAGs*. Total RNA was extracted from petals using the SparkZol Reagent (SparkJade, Shandong, China). The concentration and quality of RNA were determined using a NanoDrop 2000 (Thermo Fisher Scientific, Wilmington, DE, United States), and RNA was treated with RNase-free DNase (TaKaRa Bio Inc., Dalian, China) to eliminate traces of DNA. Afterward, complementary DNAs were synthesized using HiScript III RT SuperMix (Vazyme, Nanjing, China). By using specific primers (Supplementary Table 1), qPCR was performed using a Mastercycler ep^®^ *realplex* real-time PCR system (Eppendorf, Hamburg, Germany) with 2 × SYBR Green qPCR Mix (SparkJade, Shandong, China). Relative expression levels were calculated using the 2^–ΔΔCT^ method (Livak and Schmittgen, 2001) and presented as values relative to the control samples (0 days) after the normalization with the transcript levels of an internal control gene *DcActin*.

### Statistical Analysis

All values are means ± standard error (SE) of three independent experiments with three biological replicates for each. Data were analyzed by SPSS 22.0 software (IBM Corporation, Armonk, NY, United States). Differences among treatments were analyzed by one-way analysis of variance (ANOVA) followed by Duncan’s multiple range test or *t*-test, and *P* < 0.05 or 0.01 were considered as statistically significant.

## Results

### Characterization of Magnesium Hydride

**FIGURE 1:**
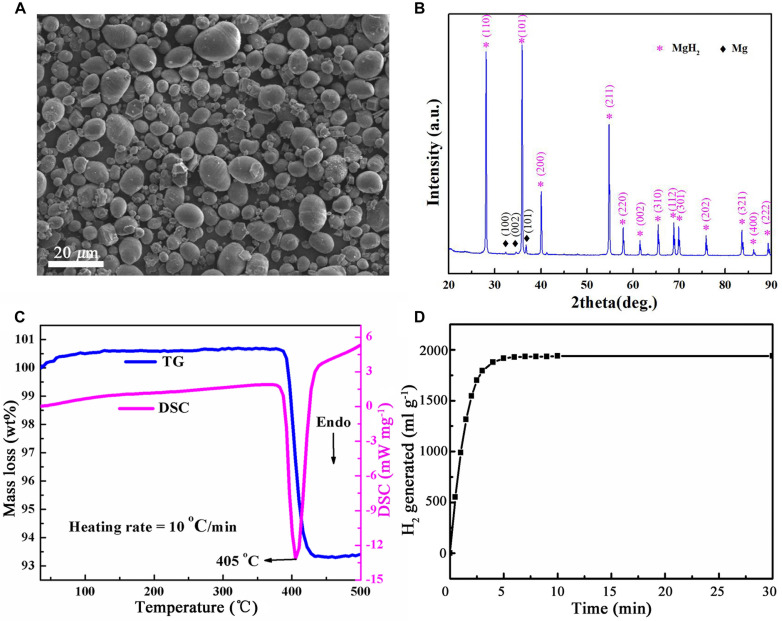
Characterization of MgH_2_ used in this work. **(A)** Scanning electron microscopy (SEM) micrographs of MgH_2_ (Scale bar = 20 μm). **(B)** X-ray diffraction (XRD) pattern of MgH_2_ powers. **(C)** Thermogravimetric (TG) and differential scanning calorimetry (DSC) curves of MgH_2_. **(D)** H_2_ generated from hydrolysis of MgH_2_.

As shown in the scanning electron microscopy images (Figure 1A), the as-received MgH_2_ particles are spherical with a diameter of 0.5–25 μm (mean diameter = 15 μm; Ma et al., 2019). The X-ray diffraction patterns (Figure 1B) confirmed that MgH_2_ is the majority phase with a small amount of unhydrided magnesium. The dehydriding properties of MgH_2_ were investigated by using differential scanning calorimetry and thermogravimetry. It was observed that the peak temperature of decomposition is 405°C at a heating rate of 10°C min^–1^ with a mass loss of about 7.2 wt% (Figure 1C).

The amount of H_2_ generated from complete hydrolysis of MgH_2_ was about 1,800 ml g^–1^ (Figure 1D); namely, the concentration of H_2_ in unit volume (1 m^3^) was 0.18% (v v^–1^). It is not flammable and explosive when the H_2_ concentration is less than 4% by volume (lower flammability limit of H_2_). Thus, it is generally safe by using MgH_2_ as a vase regent.

### Magnesium Hydride–Citrate Buffer Solution Prolongs the Vase Life of Cut Carnation Flowers

**FIGURE 2:**
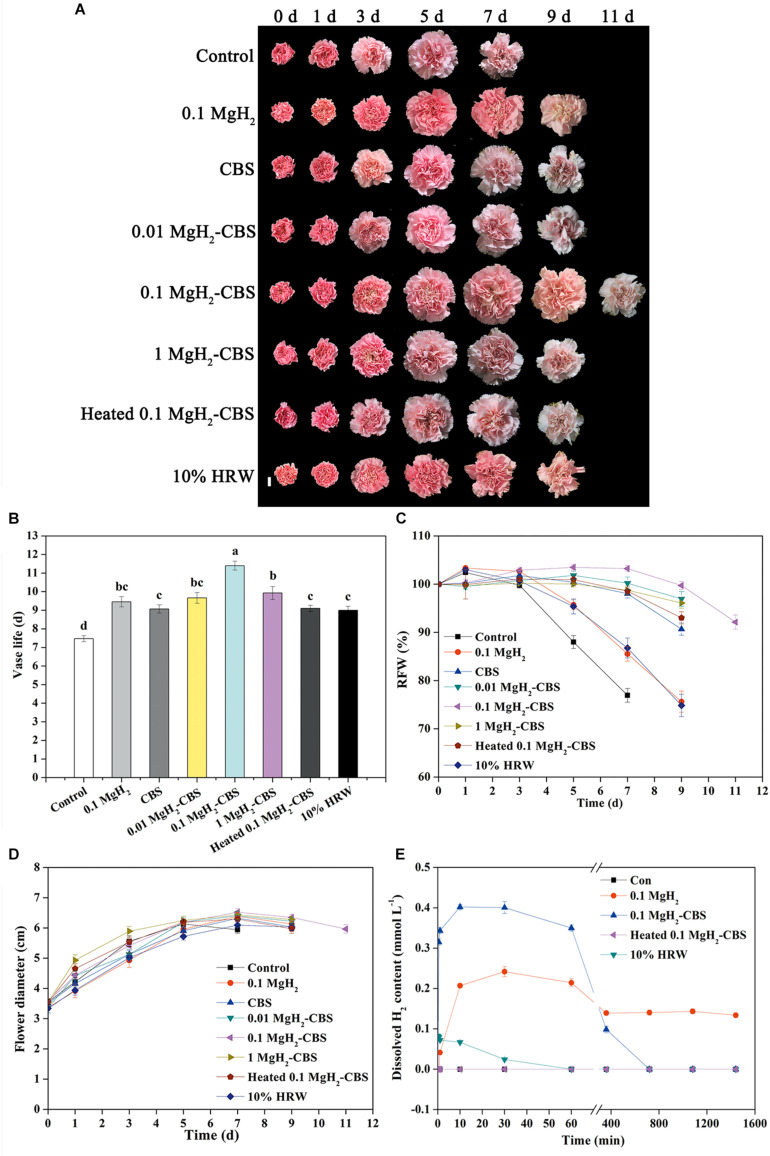
Changes in vase life, relative fresh weight (RFW), and flower diameter of cut carnations and dissolved H_2_ in solution subjected to MgH_2_, citrate buffer solution (CBS), MgH_2_-CBS, heated MgH_2_-CBS, and hydrogen-rich water (HRW). **(A)** Representative photographs of cut flowers (scale bar = 2 cm). Cut flower stems were incubated in untreated (control) and treatment solutions containing 0.1 g L^–1^ MgH_2_, 0.1-M CBS (pH 3.4) with or without 0.01, 0.1, and 1 g L^–1^ MgH_2_, 10% electrolytic HRW during vase period. Afterward, vase life **(B)**, RFW **(C)**, maximum flower diameter **(D)**, and H_2_ content in solutions **(E)** were expressed as mean ± standard error (SE). There were three replicates and 10 flowers per each for **(A–D)**, and three replicates per each for **(E)**. Experiments were conducted for three times. Bars with different letters are significantly different (*P* < 0.05), as determined by Duncan’s multiple range test.

In our experimental conditions, when 0.1 g L^–1^ MgH_2_ was dissolved in 0.1-M CBS (pH 3.4), this combined treatment (also abbreviated as MgH_2_-CBS in the following experiments) was observed as the most obvious effect on prolonging the vase life of carnation cut flowers, compared with different doses of MgH_2_, various CBS, or 10% HRW alone (Supplementary Figures 1A–C and Figures 2A,B). In the presence of 0.1 g L^–1^ MgH_2_-CBS (0.1 M, pH 3.4), for example, the vase life of the fresh cut flowers was the longest among all the treatment and was 11.4 days, which prolonged 3.9 days compared with the control, which was also significantly different from the treatments of 0.1 g L^–1^ MgH_2_ (prolonged about 2.0 days), 0.1-M CBS (pH 3.4; about 1.6 days), or 10% HRW (about 1.5 days) alone. This conclusion correlates with the data from other phenotypic parameters, including RFW and flower diameter in carnation (Figures 2C,D). By contrast, the removal of H_2_ by heating solution impaired the positive effects of MgH_2_-CBS. It was also confirmed that the boiling used in our experiment was sufficient to remove H_2_ from solutions (Figure 2E), thus suggesting the function of MgH_2_-CBS is H_2_-dependent.

Consistently, the contents of dissolved H_2_ in MgH_2_-CBS and 0.1 g L^–1^ MgH_2_ solutions ranked the first and second (rapidly peaking at 0.80 and 0.48 ppm) and remained in higher levels until 6 and 12 h, respectively. Meanwhile, H_2_ existing in 10% HRW progressively decreased, just from an initial 0.16 ppm to the basal level after 6 h (Figure 2E).

### Hydrogen Sulfide Is Involved in Magnesium Hydride–Citrate Buffer Solution-Prolonged Vase Life of Cut Carnation Flowers

**FIGURE 3:**
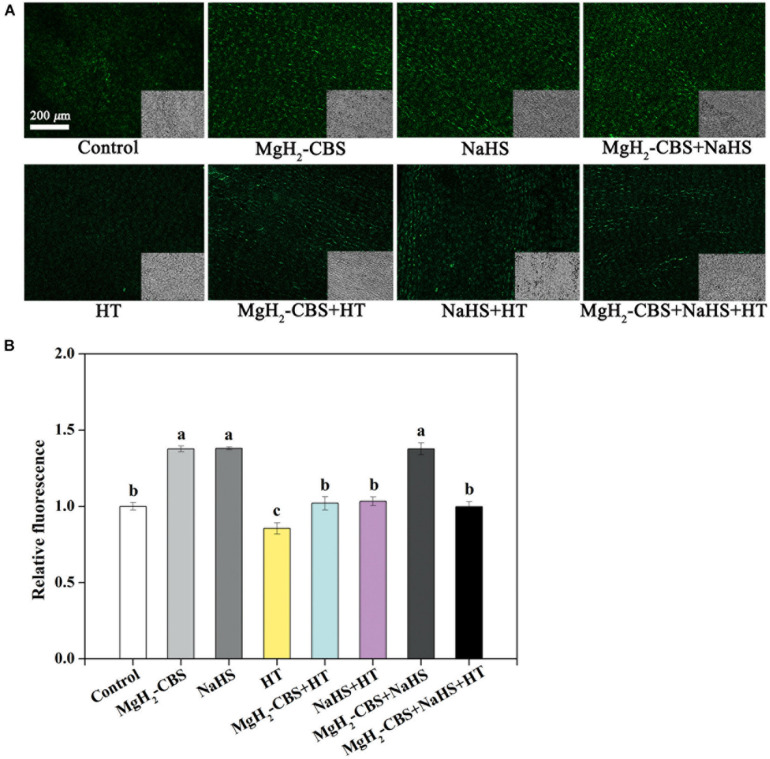
MgH_2_-CBS triggers H_2_S accumulation. **(A)** Cut flower stems were incubated in untreated (control) and treatment solutions containing 0.1 g L^–1^ MgH_2_-CBS, 600-μM NaHS, 10-mM HT (a scavenger of H_2_S), alone or their combinations for 3 days. Afterward, epidermis of petals was loaded with 20-μM WSP5 (an H_2_S fluorescent probe) and detected by laser scanning confocal microscopy (scale bar = 200 μm). Bright-field images corresponding to the fluorescent images were at the bottom right corner. **(B)** Relative fluorescence was also presented as values relative to control. Mean and SE values were calculated. At least five sections per sample were determined, and three samples in each treatment were used. Bars with different letters denoted significant differences in comparison with control at *P* < 0.05, according to Duncan’s multiple range test.

To investigate whether H_2_S is involved in MgH_2_-CBS-prolonged vase life of carnation cut flowers, both MgH_2_-CBS and HT (a specific H_2_S scavenger; Ortega et al., 2008) were applied alone and in combination. Meanwhile, NaHS (an H_2_S releasing compound) was used as a positive control. The response of the endogenous H_2_S level in the petal was monitored by labeling H_2_S using an H_2_S-specific fluorescent probe (WSP-5; Peng et al., 2014) and imaging by laser scanning confocal microscopy (Kou et al., 2018). As shown in Figure 3, the WSP-5-dependent fluorescent intensity was increased by NaHS but was greatly impaired by HT. In addition, HT alone decreased fluorescent intensity in comparison with the chemical-free control. It was confirmed that some, if not most, of the WSP-5-related fluorescence is caused by H_2_S. Further results demonstrated that MgH_2_-CBS significantly increased endogenous H_2_S production. Consistently, the inducing effect achieved by MgH_2_-CBS could be prevented by HT. Moreover, there was no additive response in fluorescence when MgH_2_-CBS was added together with NaHS.

**FIGURE 4:**
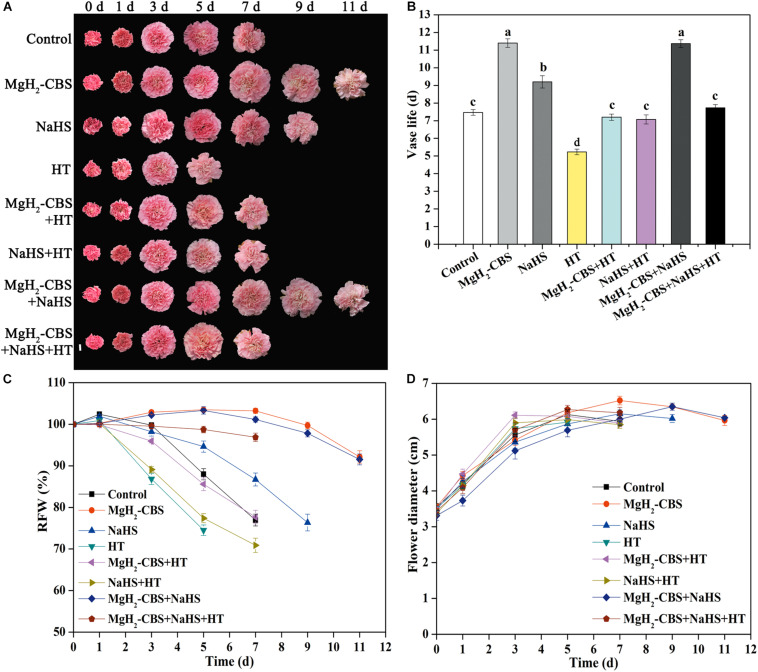
MgH_2_-CBS-prolonged vase life of cut carnation flowers is sensitive to the scavenger of H_2_S. **(A)** Cut flower stems were incubated in untreated (control) and treatment solutions containing 0.1 g L^–1^ MgH_2_-CBS, 600-μM NaHS, 10-mM HT (a scavenger of H_2_S), alone or their combinations throughout the vase period. Representative photographs of cut flowers were taken (scale bar = 2 cm). Vase life **(B)**, relative fresh weight (RFW; **C**), and maximum flower diameter **(D)** were expressed as mean and SE values. There were three replicates and 10 flowers per each. Experiments were conducted for three times. Bars with different letters are significantly different (*P* < 0.05), as determined by Duncan’s multiple range test.

The subsequent experiment was to assess the contribution of H_2_S in prolonging carnation vase-life achieved by MgH_2_-CBS. Consistently, three parameters, in terms of vase life, RFW, and flower diameter, were used. As expected, compared with the responses of NaHS, the prolonged vase life of cut carnation flowers was intensified in the presence of MgH_2_-CBS, which was abolished when HT was added (Figure 4). In contrast, compared with control, HT alone shortened the vase life. However, MgH_2_-CBS co-treated with NaHS cannot result in an additive extension of carnation vase-life. Correlating with the changes in endogenous H_2_S production (Figure 3), the results indicated that endogenous H_2_S might participate in MgH_2_-CBS-prolonged the vase life of cut carnation flowers.

### Magnesium Hydride–Citrate Buffer Solution Maintains Redox Homeostasis via Hydrogen Sulfide

**FIGURE 5:**
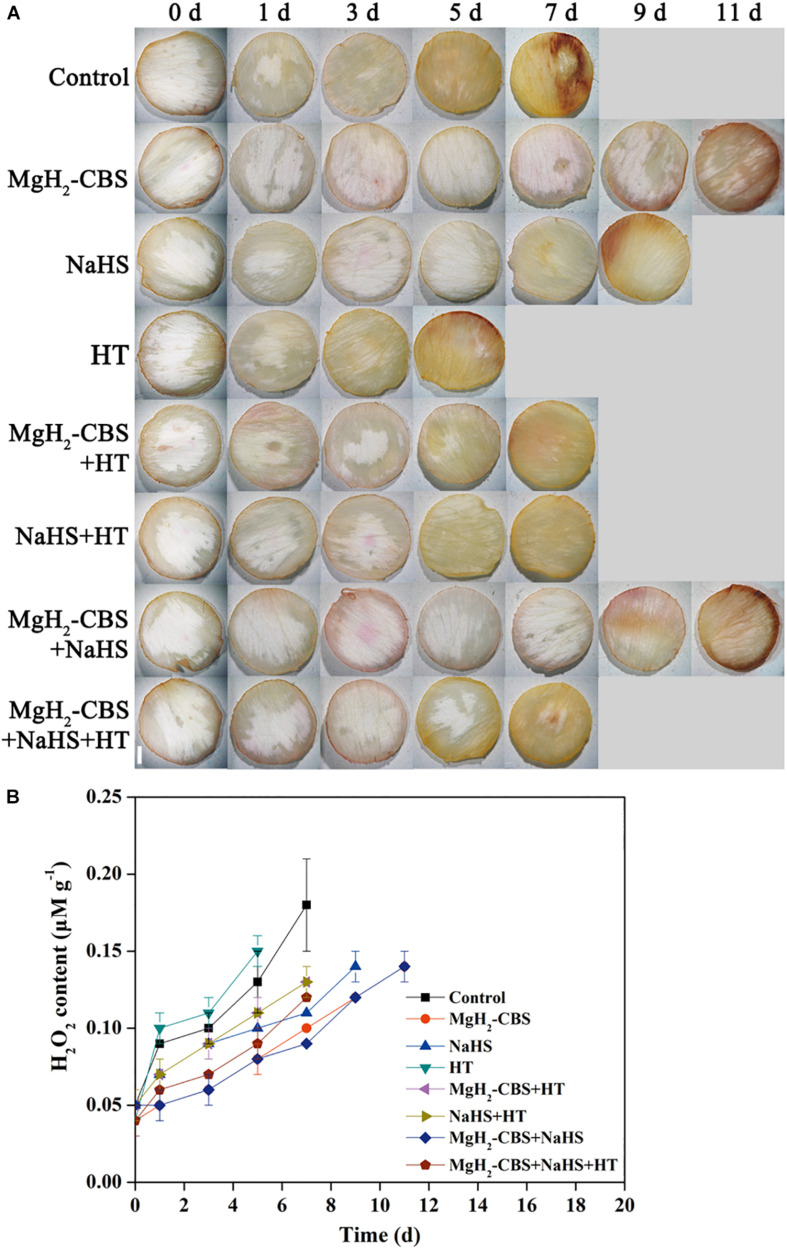
MgH_2_-CBS maintains redox homeostasis via H_2_S. **(A)** Cut flower stems were incubated in solutions containing 0.1 g L^–1^ MgH_2_-CBS, 600-μM NaHS, 10-mM HT, alone or their combinations throughout the vase period. The petals were stained with 3,3-diaminobenzidine (DAB), then photographed under a light microscope (scale bar = 1 mm). **(B)** Spectrophotography also determined H_2_O_2_ contents. Values are mean ± SE of three independent experiments with three replicated for each.

Histochemical staining of ROS (H_2_O_2_) accumulation was then adopted to reveal the detailed mechanism underlying MgH_2_-CBS-prolonged carnation vase-life. As expected, it was observed that a gradual increase of 3,3-diaminobenzidine-dependent staining in the control during the vase period (Figure 5A). The change of endogenous H_2_O_2_ level determined with spectrophotography displayed a similar tendency (Figure 5B), indicating that redox homeostasis was disrupted during senescence.

Compared with the control, the treatments with MgH_2_-CBS and NaHS individually resulted in slight staining patterns (Figure 5A). By contrast, the mentioned responses elicited by MgH_2_-CBS, and NaHS was reversed by the removal of endogenous H_2_S when HT was applied. Alone, HT brought out extensive straining compared with the control (5 days). No additive responses were observed in MgH_2_-CBS plus NaHS. Meanwhile, changes in endogenous H_2_O_2_ contents showed similar patterns (Figure 5B). These results suggested that MgH_2_-CBS could reestablish redox homeostasis in carnation flowers, which might be mediated by H_2_S.

### Role of Hydrogen Sulfide in Magnesium Hydride–Citrate Buffer Solution-Modulated Senescence-Associated Genes During Postharvest Senescence

**FIGURE 6:**
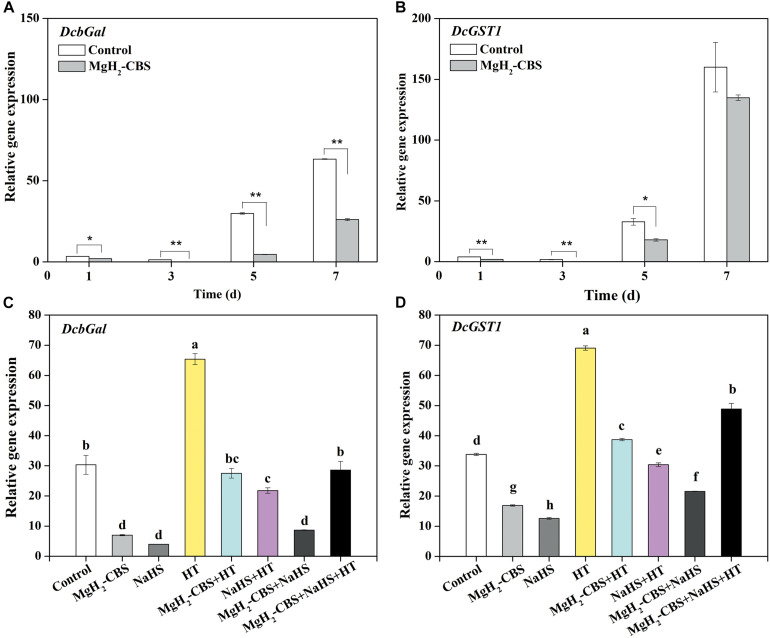
Changes in the transcripts of senescence-associated genes. Cut flower stems were incubated in solutions containing 0.1 g L^–1^ MgH_2_-CBS, 600-μM NaHS, 10-mM HT, alone or their combinations throughout the vase period. After treatments for the indicated time points or 5 days, the transcript levels of *DcbGal* **(A,C)** and *DcGST1* **(B,D)** in petals were analyzed by qPCR and presented as values relative to the control samples (0 days) after the normalization with the transcript levels of an internal control gene *DcActin*. Values are mean ± SE of three independent experiments with three replicated for each. Bars with asterisks were significantly different in comparison with control at *P < 0.05 and **P < 0.01 according to *t*-test. Bars with different letters are significantly different (*P* < 0.05), as determined by Duncan’s multiple range test.

To further elucidate the molecular mechanism of how H_2_S is involved in MgH_2_-CBS-prolonged carnation vase-life, several molecular probes responsible for senescence, including *DcbGal* and *DcGST1*, were analyzed by qPCR. The time-course experiment showed that the expression levels of *DcbGal* and *DcGST1* were increased during postharvest senescence, and those in petals of control were much higher than those in the presence of MgH_2_-CBS (Figures 6A,B). Similar to the responses of H_2_S, MgH_2_-CBS could also downregulate the transcripts of *DcbGal* and *DcGST1* (5 days; Figures 6C,D). In contrast, the inhibition mentioned earlier was attenuated by the depletion of H_2_S with HT. Additionally, HT alone could greatly increase the expression levels of these two genes. No additive inhibition responses occurred in co-treatment of MgH_2_-CBS and H_2_S as well. Therefore, H_2_S was involved in MgH_2_-CBS-induced reduction of *DcbGal* and *DcGST1* expression in carnation during the vase period.

## Discussion

At present, HRW is a major route of H_2_ administration (Shen and Sun, 2019). Ample evidence showed that HRW has positive effects on postharvest physiology. For example, HRW can prolong the shelf life (Hu et al., 2014) and decrease nitrite accumulation of fruits during storage (Zhang et al., 2019), as well as prolong the vase life of cut flowers (Ren et al., 2017; Su et al., 2019; Wang et al., 2020). Importantly, the HRW is presently mainly obtained by water electrolysis, which requires a hydrogen gas generator. Moreover, the solubility of H_2_ in water is very low (approximately 1.84 ml in 100-g H_2_O at 20°C, 1 atm; Safonov and Khitrin, 2013), and especially, the residence time of H_2_ in HRW is shorter, as the half-time of dissolved H_2_ in HRW is less than 1 h (Figure 2E), at least under our experimental conditions. The discussed disadvantages may restrict the practical applications of the electrolytic produced HRW.

In this study, H_2_ was generated by MgH_2_ hydrolysis, which was intensified when dissolved in CBS. Additionally, it can remain in higher amounts of dissolved H_2_ over a relatively longer period than the electrolytic HRW (Figure 2E). It has been reported that hydrolysis of magnesium particles can produce hydrogen nanobubbles that can exist in the water solution of a dietary supplement for a sufficiently long time (Bunkin et al., 2009; Safonov and Khitrin, 2013). A balance between surface tension and repulsive forces between surface electric charges is responsible for the stabilization of nanobubbles (Bunkin et al., 2009). We also found that the dissolution of MgH_2_ in water and CBS (in particular) was accompanied by a large number of small bubbles in the first 1–2 min. Thus, MgH_2_ may also produce hydrogen nanobubbles that increase the solubility and the residence time of H_2_. However, the dissolution of MgH_2_ in water led to a strongly alkaline environment (approximately pH 10; Supplementary Figure 1F). By contrast, the administration with CBS significantly accelerated the reaction of MgH_2_ hydrolysis and increased H_2_ generation (Figure 2E) by decreasing the pH, which is consistent with the previous studies (Hiraki et al., 2012; Chao, 2018).

It is worth noting the safety of MgH_2_ use. In fact, the concentration of H_2_ generated from MgH_2_ hydrolysis is far less than the lower flammability limit of H_2_ (4% in air). Therefore, it is safe by using MgH_2_ as a vase regent. It has been reported that the citric acid buffered around pH 3 can effectively prolong the vase life of cut flowers by reducing bacterial growth and maintaining the water balance (van Doorn, 2010). A similar result was observed in this study (Supplementary Figures 1B,C and Figures 2A,B). Although the combination of MgH_2_ and acid solutions is impractical for industry application because it causes equipment corrosion, it precisely favors postharvest preservation. We also observed that combining MgH_2_ with CBS may produce additive or synergistic effects in prolonging the vase life of cut carnation flowers. Together, MgH_2_ might be used as a promising chemical for producing a hydrogen-rich solution in horticulture.

H_2_S is a well-known important gaseous signaling molecule involved in plant developmental and environmental responses, such as root organogenesis, response to abiotic stresses, and delayed senescence of vegetables, fruits, and flowers (Zhang et al., 2011; Li et al., 2012, 2013; Wang et al., 2012; Ali et al., 2019; Corpas, 2019; Mei et al., 2019). It has been confirmed that L-cysteine desulfhydrase-dependent H_2_S acts as the downstream signal molecule involved in NO-induced heat tolerance of maize seedlings (Li et al., 2013) and methane-induced tomato and *Arabidopsis* lateral root formation (Mei et al., 2019). Interestingly, a similar requirement of H_2_S for MgH_2_-prolonged vase life of cut carnation flowers was discovered in this work. The conclusion is supported by the following pharmacologic and molecular evidence.

HT, a scavenger of H_2_S (Ortega et al., 2008; Fang et al., 2014; Mei et al., 2019), was used in our experiments, and its inhibitory role was confirmed. The increase in endogenous H_2_S accumulation triggered by MgH_2_-CBS was observed to be sensitive by HT (Figure 3). Correlating with the changes in the phenotypes of vase life, relative fresh weight, and flower diameter (Figure 4), the results presented here further revealed a requirement for endogenous H_2_S in MgH_2_-CBS-prolonged carnation vase-life.

Furthermore, ROS (especially H_2_O_2_) has been observed to increasingly produce during the senescence process in cut flower (Hossain et al., 2006; Kumar et al., 2007; Su et al., 2019). It has been demonstrated that H_2_S could inhibit ROS overproduction by increasing activities of antioxidant enzymes (Zhang et al., 2011; Hu et al., 2012, 2015). In this study, the contents of H_2_O_2_ gradually increased during the normal senescence of cut carnation flowers, which indicated the disruption of redox homeostasis (Figure 5B). The lower H_2_O_2_ levels maintained by MgH_2_-CBS might be, at least partially, responsible for delaying senescence. By contrast, the discussed responses of MgH_2_-CBS were reversed by the removal of endogenous H_2_S with HT (Figure 5B). Changes in histochemical staining showed a similar pattern (Figure 5A). The discussed results, therefore, confirmed that MgH_2_-CBS-reestablished redox homeostasis was closely associated with the alteration in endogenous H_2_S.

Recent evidence proved that H_2_S decreased the expression levels of *SAGs*, resulting in delaying the postharvest senescence of broccoli (Li et al., 2014). Furthermore, sucrose and silver thiosulphate (an inhibitor of ethylene receptor) could repress the upregulation of *SAGs* (including *DcbGal* and *DcGST*) in petals of carnation (Hoeberichts et al., 2007). Similarly, our further molecular data revealed that MgH_2_-CBS could downregulate the expression of *DcbGal* and *DcGST* (Figure 6). By contrast, such inhibition effects of MgH_2_-CBS were alleviated by HT. Combined with the changes in phenotypes and endogenous H_2_S level (Figures 3, 4), we also speculated that *SAGs* might be the target genes responsible for MgH_2_-CBS-triggered H_2_S-prolonged vase life of cut flowers.

**FIGURE 7:**
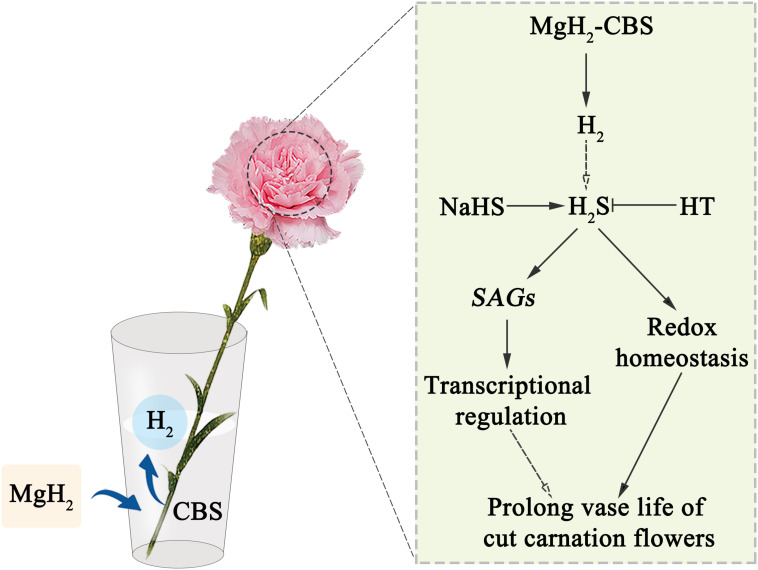
Schematic model summarizing the MgH_2_-CBS-prolonged the vase life of cut carnation flowers. CBS, citrate buffer solution; HT, hypotaurine; H_2_, hydrogen gas; H_2_S, hydrogen sulfide; MgH_2_, magnesium hydride; MgH_2_-CBS, magnesium hydride dissolved in citrate buffer solution; NaHS, sodium hydrosulfide; *SAGs*, senescence-associated genes.

Accordingly, a schematic model shown in Figure 7 summarizes the role of H_2_S in the MgH_2_-CBS-prolonged the vase life of cut carnation flowers.

## Conclusion

This study revealed the effectiveness of MgH_2_-mediated H_2_ sustainable supply in postharvest preservation of cut flowers. Compared with hydrogen-rich water, the utilization efficiency of MgH_2_ was improved by buffering with CBS. Thus, MgH_2_ may have great potential for application in horticulture. In addition, it also demonstrated a vital role of H_2_S in MgH_2_-CBS-prolonged the vase life of cut flowers by modulating the expression of *SAGs*.

## Data Availability Statement

All datasets generated for this study are included in the article/Supplementary Material, further inquiries can be directed to the corresponding author.

## Author Contributions

WS and LL conceived and designed the research. LL, YL, and SW performed the experiments and analyzed the data. JZ and WD provided advice and materials for these experiments. LL, YL, SW, and WS wrote and revised the manuscript. All authors contributed to the article and approved the submitted version.

## Conflict of Interest

The authors declare that the research was conducted in the absence of any commercial or financial relationships that could be construed as a potential conflict of interest.
