# Supplementary material for: Magnesium Hydride-Mediated Sustainable Hydrogen Supply Prolongs the Vase Life of Cut Carnation Flowers via Hydrogen Sulfide
Source: Front Plant Sci. 2020 Dec 9;11:595376. doi: 10.3389/fpls.2020.595376 (PMC7755932; doi:10.3389/fpls.2020.595376)
Supplement: Supplementary file 1 [file Data_Sheet_1.pdf]

SUPPLEMENTARY MATERIAL

Title:

Magnesium Hydride-Mediated Sustainable Hydrogen Supply Prolongs the Vase Life of Cut Carnation Flowers via Hydrogen Sulfide

*Longna Li [1], Yuhao Liu [1], Shu Wang [1], Jianxin Zou [2], Wenjiang Ding [2] and Wenbiao Shen [1,2]**

*Correspondence: Wenbiao Shen

E-mail: wbshenh@njau.edu.cn

TABLE S1 Primer sequences used for detecting senescence-associated genes (*SAGs*) by qPCR

Gene	Accession number	Primer sequence (5’-3’)
DcGST1	L05915	Forward: AGATCATAGCATCAATCACGG<br>Reverse: CGACAAGAATACTTCACGTCG
DcbGal	CF259486	Forward: AGCTTTAGATTTGGGCAGCA<br>Reverse: AGTAGATTTCCACGCGGTTG
DcActin	AY007315	Forward: GCACGGTATCGTCACCAACT<br>Reverse: AGCCTTTGGGTTAAGAGGCG

FIGURE S1

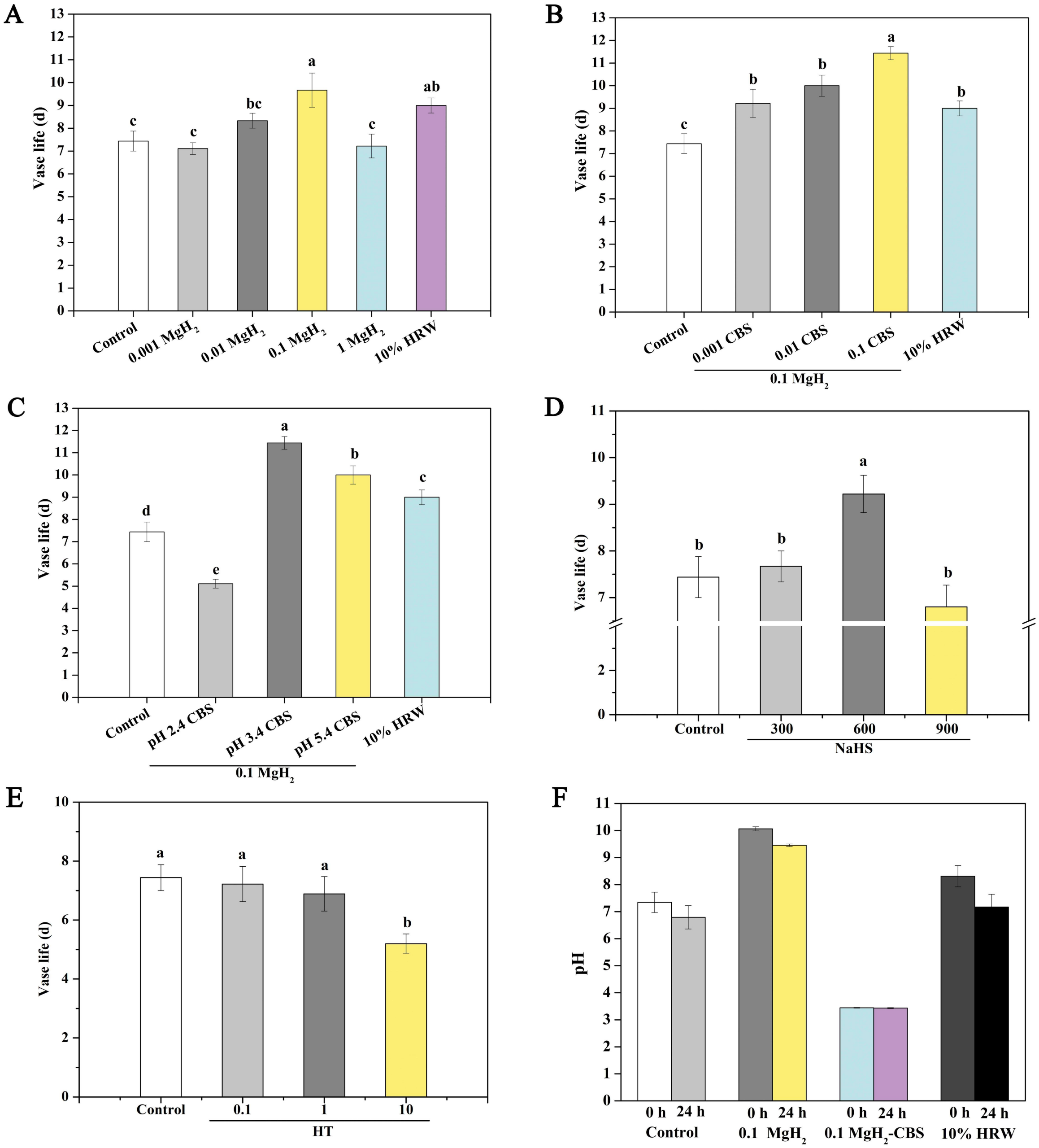

FIGURE S1 Changes in vase life of cut carnations and pH. The cut flower stems were incubated in untreated (control) and treatment solutions containing 0.001, 0.01, 0.1, and 1 g L^{-1} MgH_2, 10% HRW, 0.001, 0.01, and 0.1 M citrate buffer solution (CBS, pH 3.4) plus 0.1 g L^{-1} MgH_2, 0.1 M CBS at pH 2.4, 3.4, and 5.4 plus 0.1 g L^{-1} MgH_2, 300, 600, and 900 μM NaHS, 0.1, 1, 10 mM hypotaurine (HT) during vase period. Afterwards, vase life (**A-E**) and pH (**F**) in solutions were

recorded. Values are mean ± SE of three independent experiments with three replicated for each. Bars with different letters are significantly different ($P < 0.05$), as determined by Duncan's multiple range test.
